# Relation between Resonance Parameters of Surface Plasmon-Polariton Waves with Properties of the Dielectric-Metal Film-Dielectric Waveguide

**DOI:** 10.3390/ma13132989

**Published:** 2020-07-05

**Authors:** Stefano Bellucci, Volodymyr Fitio, Andriy Bendziak, Iryna Yaremchuk, Yaroslav Bobitski

**Affiliations:** 1 INFN-Laboratori Nazionali di Frascati, 00044 Frascati, Italy; 2 Department of Photonics, Lviv Polytechnic National University, S. Bandera str. 12, 79013 Lviv, Ukraine; v.m.fitio@gmail.com (V.F.); benzem4@gmail.com (A.B.); iryna.y.yaremchuk@lpnu.ua (I.Y.); yaroslav.v.bobytskyi@lpnu.ua (Y.B.); 3 Department of Physics, University of Rzeszów, 35959 Rzeszów, Poland

**Keywords:** prism, resonance, surface plasmon–polariton wave, spectral and angular sensitivities

## Abstract

The resonant excitation of the surface plasmon–polariton waves by the prism structure, where a thin silver film was coated on the prism, was studied. New analytical relations between the angular and spectral sensitivities on the change of the medium refractive index, adjacent to the metal film, were obtained. In addition, the analytical relation between the full width at the half maximum of the spectral and angular resonance dependencies were found.

## 1. Introduction

Biochemical reactions occurring in liquid solutions affect the refractive index of the solution itself. Knowing changes in the refractive index make it possible to determine the course of reactions and the presence of an active biological substance in the solution [1,2]. It is important in pharmaceuticals, biology and other applications in the field of biochemistry. The prism structure, consisting of a glass prism covered by a gold or silver thin film, has become of considerable use for various measurements [3,4]. In order to achieve the total internal reflection, the refractive index of the prism material must be higher than the refractive index of the researched liquid.

Therefore, prism structures of this type have been intensively studied and improved. It is proposed to coat a thin dielectric perforated layer on the metal film [5]. Thus, both the surface plasmon–polariton waves and the waveguide modes could be excited under TE (Transverse Electric) and TM (Transverse Magnetic) polarizations. The sensitivity increased due to the perforation of the dielectric waveguide layer. A thorough analysis of the structure with a dielectric layer, but without perforation, was performed in [6]. It was shown that the sensitivity of such structure is lower than the sensitivity of the classical one. In addition, it is found that the sensitivity increases when refractive index of the prism material decreases. In [7], a comparison of the sensitivity between the phase and intensity detection in the surface plasmon resonance were studied. It is proposed to arranged a thin dielectric layer with a refractive index lower than the refractive index of the prism material between the prism and the metal film [8]. In this case, the spectral and angular dependence of the reflection coefficient has two resonances, narrow and wide. The narrow resonance band is observed at a shorter wavelength. However, such an innovation did not increase the sensitivity of the sensor. It should be noted, that it is possible to increase the accuracy of detecting the values of the angles or wavelengths at which the reflectance minima are observed. In [9], it is proposed to cover a gold film with the gold grating with a period several times smaller than the wavelength. This contributed to the enhancement in the Q factor which was found to be 3–4 times higher as compared to the conventional Kretschmann configuration.

In [10], the main characteristics of the sensors (detection speed, sensitivity, portability), based on the Otto–Kretchman optical configuration, were presented. Extensive reviews of optical sensors based on resonance in dielectric gratings and on plasmon effects have been published in [11,12]. The small series of prism-based sensors, for quite a long time, were produced [13].

Numerical calculations have shown that the resonant excitation of the surface plasmon–polariton wave occurs in the prism-based structure at carefully selected parameters, namely the refractive index of the prism material, the angle of the beam incidence, wavelength, thickness of the metal film and the refractive index of the researched medium. As a result, the reflection coefficient from the metal film can be zero. When the refractive index of the researched medium changes, the minimum of the reflection coefficient dependence shifts at the constant beam propagation angle in the prism, or the resonant incidence angle changes at the constant wavelength.

The thickness of the metal film and the dielectric constant of the metal film are particularly critical. The measured values of the metals’ dielectric constant are close, but somewhat different in the experimental studies of different authors [14,15,16]. Moreover, the dielectric constant of the metal film depends on its thickness and coating technology [17]. This all leads to a difference between the results of numerical and experimental studies. However, theoretical studies and numerical analysis make it possible to understand the physical processes of plasmon–polariton resonance in the prism structure. In addition, it allows to relate the properties of the dielectric–metal film–dielectric waveguide with the sensitivity (spectral and angular) of the prism structure on the change in the researched medium refractive index.

Therefore, our research aimed to improve the understanding of processes in the prism structures in which the surface plasmon–polariton resonance occurs. In addition, the analytical Equations relating the sensor sensitivity with the waveguide properties of the dielectric–thin metal film–dielectric waveguide were founded. This made it possible to determine the reflection coefficient from the metal film depending on the wavelength, angle of incidence on the metal film, its thickness, and other parameters of the prism structure. In our opinion, these analytical expressions that have been obtained here for the first time, will be useful for further studies in this area.

## 2. Sensitivity of Prism Structure and its Relation with Properties of the Dielectric–Metal Film–Dielectric Waveguide

**Figure 1:**
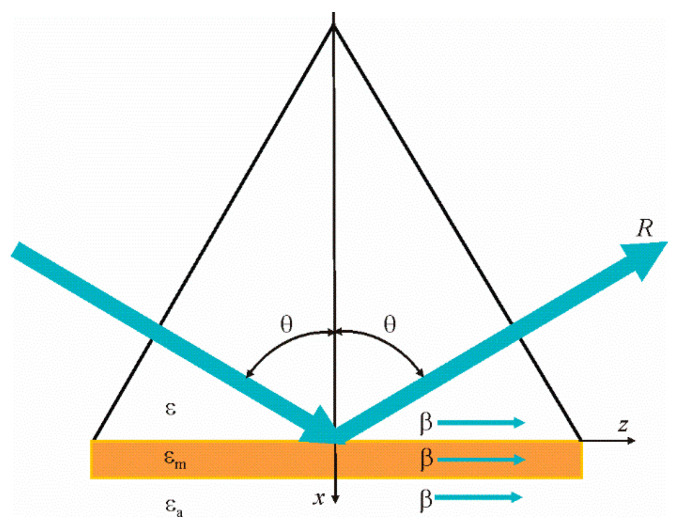
The optical scheme of the prism structure, β is propagation the constant of the surface plasmon polariton wave, ε is the dielectric constant of the prism material, $\varepsilon_{m}$ is the dielectric constant of the metal, and $\varepsilon_{a}$ is the dielectric constant of the researched medium.

A typical prism structure in which a thin gold or silver metal film is deposited based on a triangular prism is shown in Figure 1. The metal film adjoins the researched medium with the refractive index $n_{a}=\sqrt{\varepsilon_{a}}$. Plane wave propagates into the glass prism, and falls on the metal film at an angle θ and is reflected at the same angle. The reflection coefficient *R* can be zero when the surface plasmon–polariton resonance occurs in the structure.

Surface plasmon–polariton wave excited under resonance can be described by as follows:

(1) $$E\left(x,z\right)=E_{0}\left(x\right)\exp\left(i\beta z\right)$$

One of the two propagation constants β under resonance is real according to [9,18]. Thus, the following relation is true:

(2) $$\frac{2\pi n}{\lambda }\sin\theta =\beta \left(\lambda ,n_{a}\right)$$

where λ is the wavelength of incident radiation, and *n* is the prism material refractive index.

Equality in Equation (2) is strong at the resonance. Therefore, knowing the propagation constant β, one can determine the resonant incidence angle θ of the plane wave.

Propagation constant β can be determined from the following equation [19]:

(3) F(x,y)=exp(−2kmd)−kmεm+kεkmεm−kε×kmεm+kaεakmεm−kaεa=0

where *x* and *y* are real and imaginary parts of the propagation constant, respectively, that is β=x+iy, $k_{m}^{2}=\beta^{2}-k_{0}^{2}\varepsilon_{m}$, $k^{2}=\beta^{2}-k_{0}^{2}\varepsilon$, $k_{a}^{2}=\beta^{2}-k_{0}^{2}\varepsilon_{a},$ $k_{0}^{2}={\left(\frac{2\pi }{\lambda }\right)}^{2},$ *d* is metal layer thickness.

**Figure 2:**
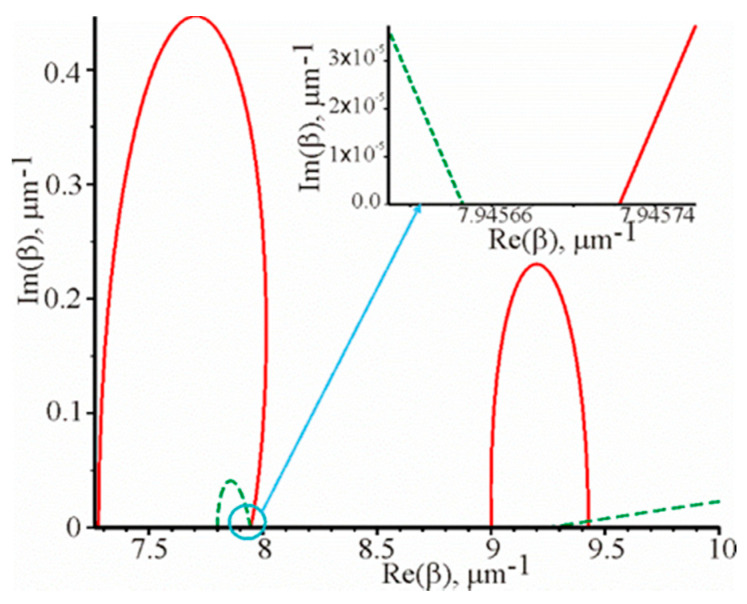
Graphical dependencies Re(F(x,y))=0 (solid line) and Im(F(x,y))=0 (dotted line) at the following parameters of the prism structure: λ=1.064 μm, θ=1.040226 rad, d=52.55 nm, $\varepsilon_{m}=-57.1915+i1.26097\;$ [16,20], n=1.56 and $n_{a}=1.3242$. The reflection coefficient from the metal film is 0.000041 at these parameters. The insertion shows solid and dotted curves on a larger scale.

Function F(x,y) is generally complex, and will be zero if the real and complex parts of this function are also zero. Therefore, the intersection of the curves corresponding to the following Equations:

(4) Re(F(x,y))=0, Im(F(x,y))=0

will determine the required propagation constants. Figure 2 shows the corresponding graphical dependencies.

These dependencies are shown in the first quadrant, when the surface plasmon–polariton wave propagates in the positive direction, as shown in Figure 1. In Equations (3) and (4), we exclusively have $\beta^{2}$. If β is a solution of these Equations, and due to the fact that $\beta^{2}\;$= ${\left(-\beta \right)}^{2}$, then –β will also be the solution of the system of Equations (4). This implies a simple physical meaning: the opposite signs before β correspond to the opposite propagation of the surface plasmon–polariton wave on the metal film. Notice that these Equations Re(*F*(x, y)) = 0, Im(*F*(x, y)) = 0 are not explicitly expressed functions, i.e., as we have no explicit y = f(x). The graphs of these functions are built using standard software, such as MAPLE, using a single operator. At the intersection of these two functions Re(*F*(x, y)) = 0, Im(*F*(x, y)) = 0 (Figure 2) we have the required values of Reβ and Imβ.

Thus, the solution of the system of Equation (4) will be in the third quadrant and the trend of the curves Re(F(x,y))=0 and Im(F(x,y))=0 will be symmetric with respect to the origin of the coordinates in the first and third quadrant. The solutions in the second and fourth quadrants will also be symmetrical with respect to the origin of the coordinates. However, they will not have physical meaning, since they provide the amplification of the surface plasmon–polariton wave during propagation. For instance, in the fourth quadrant, Re(β) > 0, Im(β) < 0, therefore, the field strength will increase as the wave propagates in the positive direction of the z axis according to Equation (1). Therefore, only Re(F(x,y))=0, and Im(F(x,y))=0 are shown for the first quadrant in Figure 2. The trend of the curves Re(F(x,y))=0 and Im(F(x,y))=0 is not symmetrical with respect to the axes Re(β) and Im(β).

Thus, it can be seen that there are the two propagation constants. One of them is real and the other is complex. The real propagation constant corresponds to the resonant excitation and the propagation of the surface plasmon–polariton wave. This propagation constant can be calculated from the left side of Equation (2) and it is equal to 7.945684 ${\mu m}^{-1}$. Moreover, it is also equal to the average coordinates of the intersection of the solid and the dotted curves with the axis Re(β), which follows from the insertion in Figure 2. In this insertion, the trend of the curves Im(F(x,y))=0 and Re(F(x,y))=0 is shown within the blue circle in Figure 2, which is shown on an enlarged scale.

Therefore, in this case, the solid curve Re(F(x,y))=0 and the dotted curve Im(F(x,y))=0 do not intersect at one point with the abscissa. It can be explained by the fact that although the reflection coefficient is close to zero, but not equal. It is possible to specify the parameters of the metal film, the wavelength and the incidence angle at which the reflection coefficient was significantly smaller than in the example described above.

**Figure 3:**
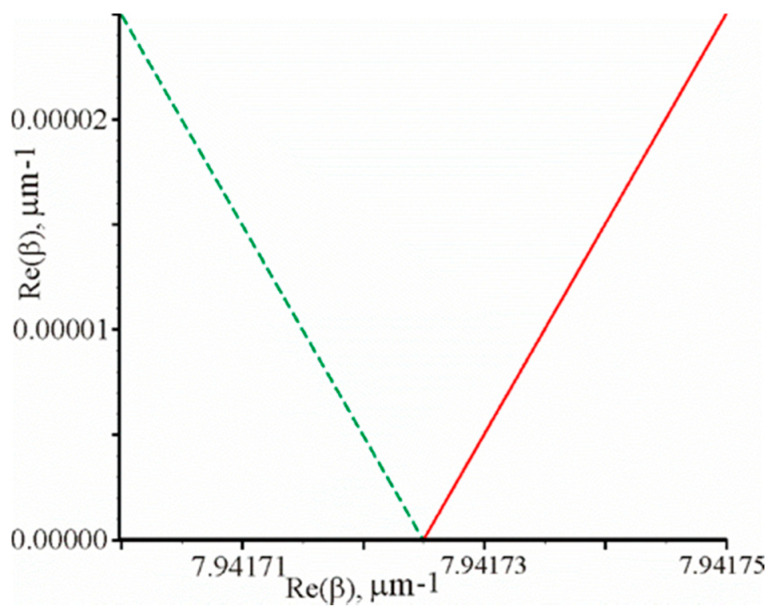
Graphical dependencies Re(F(x,y))=0 (solid line) and Im(F(x,y))=0 (dotted line) at the prism structure parameters mentioned above.

The reflection coefficient is equal to $R=2.6\times 10^{-14}$ at the following parameters: λ=1.0645076 μm, θ=1.04018937 rad, d=52.7 nm. Figure 3 shows the graphical dependencies Re(F(x,y))=0 (solid curve) and Im(F(x,y))=0 (dotted curve). It can be seen that these two curves intersect at one point on the abscissa axis. Thus, the propagation constant will be $\beta =7.94172490\;{\mu m}^{-1}.$ The propagation constant calculated by the left side of Equation (2) is equal to $\beta =\frac{2\pi n}{\lambda }\sin\theta =$ $7.94172494\;{\mu m}^{-1}.$ That is, Equation (2) is valid, since there is a good match of the calculated propagation constant by different methods.

Relations similar to Equation (2) exist under the guided-mode resonance in the dielectric gratings [21], as well as in the system of dielectric or metal grating on the metal substrate [12] under the surface plasmon–polariton resonance. However, in this case, these relations are approximate because the gratings violate the layers’ homogeneity. It should be noted, that these relations will be more accurate as the modulation coefficient of the grating dielectric constant is smaller, as shown by numerical experiments in [21].

The coefficient of the reflection of such structure was calculated by the matrix method [22]. In accordance with this method, the reflection coefficient in amplitude is determined as follows:

(5) $$r\left(d,\theta ,\lambda \right)=\frac{\eta E_{0}-H_{0}}{\eta E_{0}+H_{0}}$$

where η=ncosθ is the effective refractive index of the prism material for TM polarization waves, $E_{0}$ and $H_{0}$ are the amplitudes of the electric and magnetic field strengths in the prism, respectively.

Thus, the reflection coefficient in intensity can be written in the following form:

$$R\left(d,\theta ,\lambda \right)={\left|r\left(d,\theta ,\lambda \right)\right|}^{2}$$

In order to calculate r(d,θ,λ), it is needed to know the numerator and denominator of Equation (5), which are respectively equal to:

$$\begin{array}{cc} \eta E_{0}-H_{0}= & \left(\frac{-in_{a}\sin\left(2\pi n_{m}d\sqrt{1-n^{2}\sin{\left(\theta \right)}^{2}/n_{m}^{2}}/\lambda \right)\sqrt{1-n^{2}\sin{\left(\theta \right)}^{2}/n_{m}^{2}}}{\sqrt{1-n^{2}\sin{\left(\theta \right)}^{2}/n_{a}^{2})}n_{m}}+\cos\left(2\pi n_{m}d\sqrt{1-n^{2}\sin{\left(\theta \right)}^{2}/n_{m}^{2}}/\lambda \right)\right)\frac{n}{\cos\theta }\\ & -\frac{n_{a}\cos\left(2\pi n_{m}d\sqrt{1-n^{2}\sin{\left(\theta \right)}^{2}/n_{m}^{2}}/\lambda \right)}{\sqrt{1-n^{2}\sin{\left(\theta \right)}^{2}/n_{a}^{2}}}\\ & +\frac{i\sin\left(2\pi n_{m}d\sqrt{1-n^{2}\sin{\left(\theta \right)}^{2}/n_{m}^{2}}/\lambda \right)n_{m}}{\sqrt{1-n^{2}\sin{\left(\theta \right)}^{2}/n_{m}^{2}}},\\ \eta E_{0}+H_{0}= & \left(\frac{-in_{a}\sin\left(2\pi n_{m}d\sqrt{1-n^{2}\sin{\left(\theta \right)}^{2}/n_{m}^{2}}/\lambda \right)\sqrt{1-n^{2}\sin{\left(\theta \right)}^{2}/n_{m}^{2}}}{\sqrt{1-n^{2}\sin{\left(\theta \right)}^{2}/n_{a}^{2})}n_{m}}+\cos\left(2\pi n_{m}d\sqrt{1-n^{2}\sin{\left(\theta \right)}^{2}/n_{m}^{2}}/\lambda \right)\right)\frac{n}{\cos\theta }\\ & +\frac{n_{a}\cos\left(2\pi n_{m}d\sqrt{1-n^{2}\sin{\left(\theta \right)}^{2}/n_{m}^{2}}/\lambda \right)}{\sqrt{1-n^{2}\sin{\left(\theta \right)}^{2}/n_{a}^{2}}}\\ & -\frac{i\sin\left(2\pi n_{m}d\sqrt{1-n^{2}\sin{\left(\theta \right)}^{2}/n_{m}^{2}}/\lambda \right)n_{m}}{\sqrt{1-n^{2}\sin{\left(\theta \right)}^{2}/n_{m}^{2}}} \end{array}$$

It can be seen that the numerator and denominator have opposite signs in front of the third and fourth terms.

**Figure 4:**
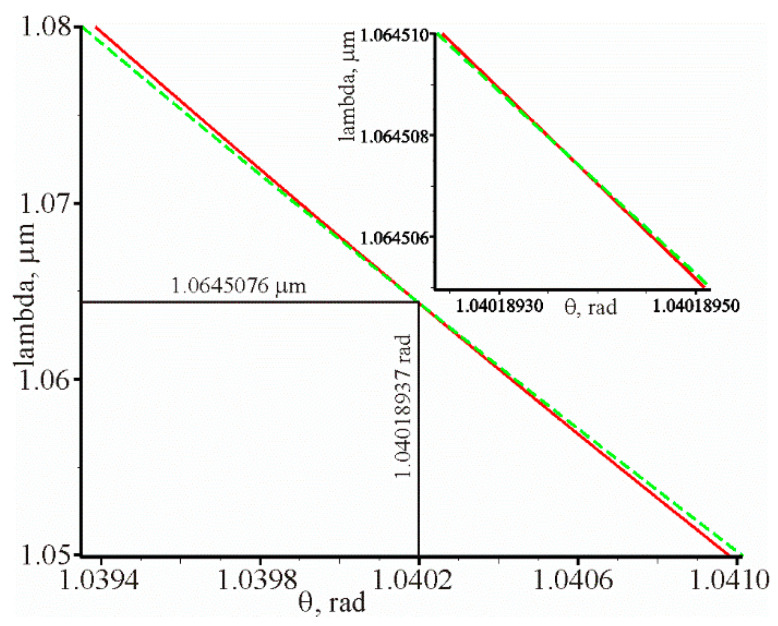
Graphical dependencies Re(r(θ,λ))=0 (solid line) and Im(r(θ,λ))=0 (dotted line) at the following parameters: d=52.69 nm, =1.56, $n_{a}=1.3242$. The intersection of the solid and dotted curves determines the θ and λ, at which the reflection coefficient is equal to $R=2.6\times 10^{-14\;}.$ The inset shows parts of the intersected curves on a larger scale.

The value of the resonant wavelength λ and the resonant incidence angle θ of the beam on the grating at other given parameters of the prism structure can be determined from the condition that the amplitude reflection coefficient is zero r(λ, θ)=0 or the numerator of Equation (5) must also be zero. The intersection of the curves Re(r(λ, θ))=0 (solid curve) and Im(r(λ, θ))=0 (dotted curve) in Figure 4 defines the values λ and θ at which the amplitude of the reflection coefficient is zero. The intersection of these curves corresponds to the values λ=1.0645076 μm, θ=1.04018937 rad. It can be seen that the solid and dotted curves (almost straight lines) intersect at a very acute angle. This means that with a slight change in some parameter of the structure, the resonance will not brake and the reflection coefficient will be close to zero.

Equation (2) is accurate under resonance. It can be seen that the left side of this Equation in the measurement process can depend on the angle θ, and the right part depends on the wavelength λ and the researched medium refractive index $n_{a}$. On the basis of this Equation (2) it is possible to establish a relation between the parameters of the waveguide, which are present in the right-hand side of the Equation, and the characteristics of the sensor as a whole.

Let the angle of incidence be fixed, and the resonant wavelength will change by dλ changing $n_{a}$ by $dn_{a}$. Accordingly, the change of the right and left parts of Equation (2) can be written as follows:

(6) $$-\frac{2\pi n}{\lambda^{2}}\sin\theta \;d\lambda =\frac{\partial \beta }{\partial n_{a}}dn_{a}+\frac{\partial \beta }{\partial \lambda }d\lambda$$

Let us introduce the following notation $S_{n}^{\left(\beta \right)}=\frac{\partial \beta }{\partial n_{a}},$ $S_{\lambda }^{\left(\beta \right)}=\frac{\partial \beta }{\partial \lambda }.$ From Equation (5) after simple algebraic transformations, taking into account Equation (2), one can find the spectral sensitivity as the ratio of the resonant wavelength change and the change of the researched medium refractive index, as follows::

(7) $$S_{\lambda }=\frac{d\lambda }{dn_{a}}=-\frac{S_{n}^{\left(\beta \right)}\lambda }{S_{\lambda }^{\left(\beta \right)}\lambda +\beta }$$

The resonant angle θ will change at the fixed wavelength when the refractive index of the researched medium changes. On the basis of Equation (2) the following Equation was obtained:

(8) $$\frac{2\pi n}{\lambda }\cos\theta \;d\theta =\frac{\partial \beta }{\partial n_{a}}dn_{a}$$

From Equation (8) it is possible to find the angular sensitivity, as the ratio of the resonant angle change and the change in the researched medium refractive index, as follows:

(9) $$S_{\theta }=\frac{d\theta }{dn_{a}}=\frac{S_{n}^{\left(\beta \right)}\lambda }{2\pi n\cos\theta }$$

On the basis of Equation (2), with a constant refractive index $n_{a}$, it can be found the change of the resonant wavelength by δλ as the corresponding incidence angle changes by δθ using the following Equation:

(10) $$-\frac{2\pi n}{\lambda^{2}}\sin\theta \;\delta \lambda +\frac{2\pi n}{\lambda }\cos\theta \;\delta \theta =\frac{\partial \beta }{\partial \lambda }\delta \lambda$$

In fact, based on Equation (10), it is possible to calculate how full the width at the half maximum of the spectral and angular resonance dependencies are, related in the next form:

(11) $$\delta \lambda_{0.5}=-\frac{2\pi n\cos\theta }{S_{\lambda }^{\left(\beta \right)}\lambda +\beta }\delta \theta_{0.5}$$

Let us divide the right part of Equation (7) by the right part of Equation (11), and as a result will be obtained:

(12) $$\frac{S_{\lambda }}{\delta \lambda_{0.5}}=\frac{-\frac{S_{n}^{\left(\beta \right)}\lambda }{S_{\lambda }^{\left(\beta \right)}\lambda +\beta }}{-\frac{2\pi n\cos\theta }{S_{\lambda }^{\left(\beta \right)}\lambda +\beta }\delta \theta_{0.5}}=\frac{\frac{S_{n}^{\left(\beta \right)}\lambda }{2\pi n\cos\theta }}{\delta \theta_{0.5}}=\frac{S_{\theta }}{\delta \theta_{0.5}}$$

The last Equation is crucial for the sensors, since it determines the sensor suitability for measuring the change in the refractive index of the researched medium. The higher the value, the more accurate the refractive index can be measured. It is intuitively felt that Equation (12) is valid for the sensors based on resonance phenomena. However, if $\delta \lambda_{0.5}$ or $\delta \theta_{0_{0.5}}$ are too narrow, then it will be difficult to measure such narrow resonances.

Using Equation (3), the propagation constants $\beta \left(\lambda ,n_{a}\right),$ and on their basis $S_{\lambda }^{\left(\beta \right)}$ and $S_{n}^{\left(\beta \right)}$ can be determined using the following Equations:

(13) $$S_{\lambda }^{\left(\beta \right)}=\frac{\beta \left(\lambda_{rez}+\Delta \lambda ,n_{a}\right)-\beta \left(\lambda_{rez}-\Delta \lambda ,n_{a}\right)}{2\Delta \lambda }$$

(14) $$S_{n}^{\left(\beta \right)}=\frac{\beta \left(\lambda_{rez},n_{a}+\Delta n_{a}\right)-\beta \left(\lambda_{rez},n_{a}-\Delta n_{a}\right)}{2\Delta n_{a}}$$

where Δλ=0.0001 μm, $\Delta n_{a}=0.0001$ at the conditon that d=52.7 nm, n=1.56, $n_{a}=1.3242$, λ=1.0645076 μm, θ=1.04018937 rad, $S_{n}^{\left(\beta \right)}=6.18685\;\mu m^{-1}$, $S_{\lambda }^{\left(\beta \right)}=-7.7219{\;\mu m}^{-2}.$

**Figure 5:**
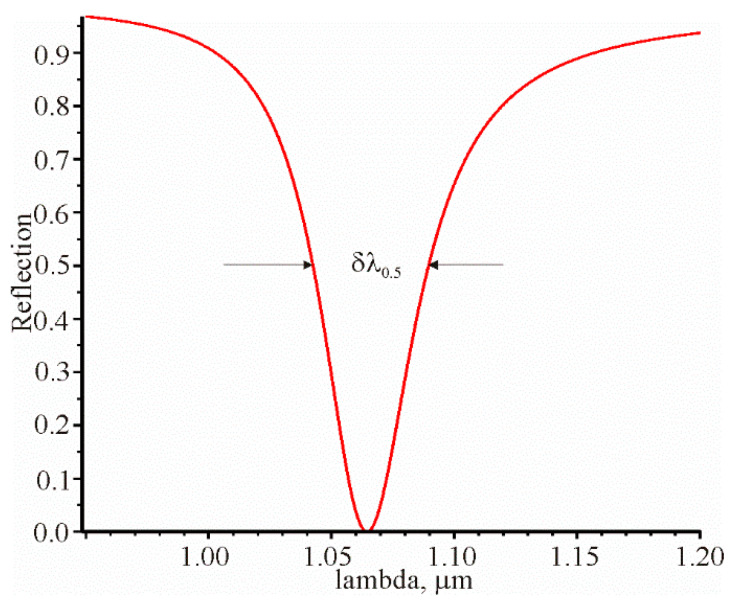
Spectral dependence of the reflection coefficient with the following parameters: $\lambda_{rez}=1.0645076\;\mu m,$ $\theta_{rez}=1.04018937,$ d=52.7 nm, n=1.56, $n_{a}=1.3242$. In this case, $\delta \lambda_{0.5}$ = 0.0471 μm.

It is necessary to have $\delta \lambda_{0.5}$ or $\delta \theta_{0_{0.5}}$ to fully describe the prism structure as a sensor. These parameters can be determined from the spectral or angular dependence of the reflection coefficient. The spectral dependence of the reflection coefficient is shown in Figure 5.

**Figure 6:**
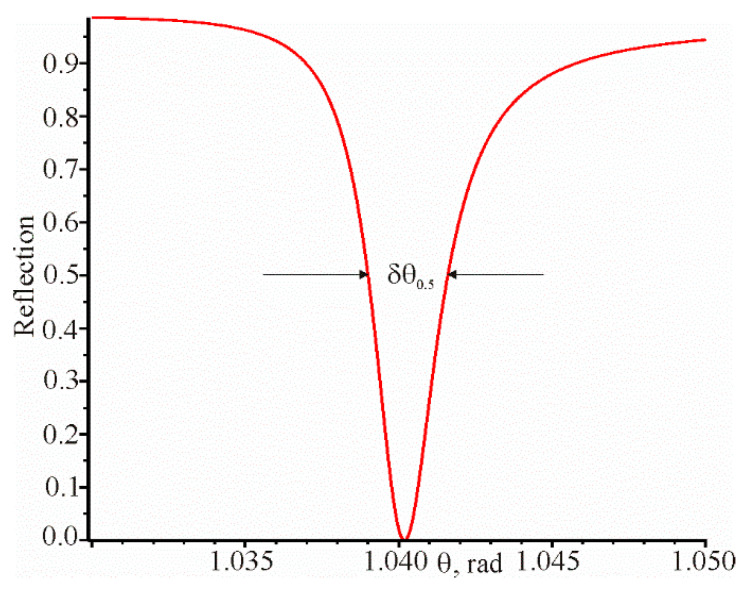
Angular dependence of the reflection coefficient with the following parameters: $\theta_{rez}=1.04018937,$ $\lambda_{rez}=1.0645076\;\mu m,$ d=52.7 nm, n=1.56, $n_{a}=1.3242$. In this case, $\delta \theta_{0.5}\;$ = 0.002609 rad.

The angular dependence of the reflection coefficient at a constant resonant wavelength is shown in Figure 6.

In order to verify the correctness of Equations (7), (9), (11) and (12), it is necessary to calculate $S_{\lambda }$. It should be calculated on the base of the resonant wavelength change at the given angle of incidence of the beam, with a slight change in the researched medium refractive index. It can be done using the following equation:

(15) $$S_{\lambda }=\frac{\lambda_{rez}\left(n_{a}+\Delta n_{a}\right)-\lambda_{rez}\left(n_{a}-\Delta n_{a}\right)}{2\Delta n_{a}}$$

Sensitivity $S_{\theta }$ at the given resonance wavelength can be determined as follows:

(16) $$S_{\theta }=\frac{\theta_{rez}\left(n_{a}+\Delta n_{a}\right)-\theta_{rez}\left(n_{a}-\Delta n_{a}\right)}{2\Delta n_{a}}$$

In both cases, at calculating sensitivities $S_{\lambda }$ and $S_{\theta }$ the $\Delta n_{a}=0.0001$. The sensitivities are $S_{\lambda }=24.08$ μm and $S_{\theta }=1.328$ rad.

The summarized results of the calculation are presented in Table 1. The numbers in brackets indicate the Equations used during the calculations, or on the basis of which figure, the full width at the half maximum of the spectral and angular resonance dependencies are determined.

Comparisons of the cells in rows 1 and 2 contained in columns 4, 5 and 6 show no significant difference between them. That is the relative difference that is not more than 1.6%. If these cells are rounded to two significant digits, then the difference between the data of the corresponding cells will be zero. It can be seen that the cells in columns 7 and 8 are almost the same. In general, the data in Table 1 confirm the correctness of the analytical relations that relate the properties of the dielectric–metal thin film–dielectric waveguide with the parameters of the prism structure as a sensor.

## 3. Conclusions

Using strong equality under resonance according to Equation (2), the simple analytical Equations that relate the waveguide parameters $S_{n}^{\left(\beta \right)}$ and $S_{\lambda }^{\left(\beta \right)}$ with the characteristics of the prism structure as a sensor, in particular: $\delta \theta_{0.5}$, $\delta \lambda_{0.5},$ $S_{\theta },\;\;S_{\lambda },$ $\frac{S_{\theta }}{\delta \theta_{0.5}}$, $\frac{S_{\lambda }}{\delta \lambda_{0.5}},$ were obtained. It is shown that the full width at the half maximum of the spectral and angular resonance dependencies are related by Equation (11). In addition, Equation (12) was obtained. It is important for the sensors since it determines the applicability of the sensor to measure the change in the researched medium refractive index. It is intuitively felt that this ratio is characteristic of other types of sensors, the work of which is based on resonance phenomena. For instance, the surface plasmon–polariton resonance in metal gratings or guided-mode resonance in the dielectric grating. The obtained analytical Equations are confirmed by the numerical calculations, based on which the angular and spectral sensitivities, as well as the widths of the resonance curves, were determined. From the obtained analytical Equation (8), it is clear that the angular sensitivity increases when the wavelength and incidence angle of the waves on a metal film increase and when the refractive index of the prism material decreases. Spectral sensitivity increases when wavelength increases.

An analytical expression is obtained that determines the reflection coefficient from the metal film depending on the wavelength, angle of incidence, its thickness and other parameters of the prism structure.

The strong equality of the right and left sides of Equation (2) under resonance is explained by the fact that the planarity of the boundaries between the metal film and the dielectrics in the prism structure is not disturbed. Similar Equations are approximate for grating structures, where the resonance of surface plasmon–polariton or guided-mode resonance occurs. It is due the fact that the relief grating disturbs the planarity of boundaries between the different media or disturbs the homogeneity of the layers by a volume dielectric grating. The angular and spectral sensitivity to the change in the refractive index of the test medium can be calculated, based on the sensitivity of the propagation constant β of the waveguide to the change in the wavelength and to the change in the refractive index.

## Figures and Tables

**Table 1:** Characteristic parameters of the waveguide ($S_{n}^{\left(\beta \right)},\;S_{\lambda }^{\left(\beta \right)}$) and the parameters of the prism structure under the excitation of the surface plasmon–polariton resonance.

Parameters	$S_{n}^{\left(\beta \right)},$ $\mu m^{-1}$	$S_{\lambda }^{\left(\beta \right)},$ $\mu m^{-2}$	$\delta \theta_{0.5},rad$	$\delta \lambda_{0.5},$ μm	$S_{\theta },\;rad$	$S_{\lambda },\;\mu m$	$\frac{S_{\theta }}{\delta \theta_{0.5}}$	$\frac{S_{\lambda }}{\delta \lambda_{0.5}}$
No.	1	2	3	4	5	6	7	8
1	6.18685 (13) *	−7.7219 (12)	0.00261 Figure 6	0.0471 Figure 5	1.33 (15)	24.08 (14)	509	511
2	6.18685 (13)	−7.7219 (12)	0.00261 Figure 6	0.0465 (10)	1.33 (8)	23.70 (6)	510	510

*The number in the parentheses is the number of the Equation used for the calculation. The two different rows show that some date are calculated by different methods to verify the correctness of the obtained expressions. The No. are only provided to identify the various columns for convenience when discussing the results illustrated in the Table 1.

## References

[1] Homola J. (2008). Surface plasmon resonance sensors for detection of chemical and biological species. Chem. Rev..

[2] Puiu M., Bala C. (2016). SPR and SPR imaging: Recent trends in developing nanodevices for detection and real-time monitoring of biomolecular events. Sensors.

[3] Kretschmann E., Raether H. (1968). Radiative decay of nonradiative surface plasmons excited by light. Z. Nat. A.

[4] Otto A. (1968). Excitation of nonradiative surface plasma waves in silver by the method of frustrated total reflection. Z. Phys. A.

[5] Awazu K., Rockstuhl C., Fujimaki M., Fukuda N., Tominaga J., Komatsubara T., Ikeda T., Ohki Y. (2007). High sensitivity sensor made of perforated waveguides. Opt. Express.

[6] Fitio V., Yaremchuk I., Bobitski Y. (2011). Optical excitation of surface plasmon polariton and waveguide modes resonances on prismatic structures. Opt. Appl..

[7] Ran B., Lipson S.G. (2006). Comparison between sensitive of phase and intensity detection in surface plasmon resonance. Opt. Express.

[8] Ayushi P., Tomar M., Gupta V. (2019). Refractive index sensor using long-range surface plasmon resonance with prism coupler. Plasmonics.

[9] Arora P., Talker E., Mazurski N., Levy U. (2018). Dispersion engineering with plasmonic nano structures for enhanced surface plasmon resonance sensing. Sci. Rep..

[10] Zeng Y., Hu R., Wang L., Gu D., He J., Wu S.Y., Shao Y. (2017). Recent advances in surface plasmon resonance imaging: Detection speed, sensitivity, and portability. Nanophotonics.

[11] Quaranta G., Basset G., Martin O.J.F., Gallinet B. (2018). Recent Advances in Resonant Waveguide Gratings. Laser Photonics Rev..

[12] Xu Y., Bai P., Zhou X., Akimov Y., Png C.E., Ang L.K., Knoll W., Wu L. (2019). Optical Refractive Index Sensors with Plasmonic and Photonic Structures: Promising and Inconvenient Truth. Adv. Opt. Mater..

[13] Snopok B.A., Kostyukevich K.V., Lysenko S.I., Lytvyn P.M., Lytvyn O.S., Mamykin S.V., Venger E.F. (2001). Optical biosensors based on the plasmon resonance phenomenon: Optimazation of the metal lauer parameters. Semicond. Phys. Quantum Electron. Optoelectron..

[14] McPeak K.M., Jayanti S.V., Kress S.J.P., Meyer S., Iotti S., Rossinelli A., Norris D.J. (2015). Plasmonic films can easily be better: Rules and recipes. ACS Photonics.

[15] Babar S., Weaver J.H. (2015). Optical constants of Cu, Ag, and Au revisited. Appl. Opt..

[16] Johnson P.B., Christy R.W. (1972). Optical Constants of the Noble Metals. Phys. Rev. B.

[17] Arsenin A.V., Stebunov Y.V., Fedyanin D.Y., Volkov V.S. (2017). Optical constants and structural properties of thin gold films. Opt. Express.

[18] Fitio V.M., Laba H.P., Bobitski Y.W. (2007). Absorption of Electromagnetic Waves into Periodic Structure and Thin Film of Metal when a Resonance of Plasmons Appears as a Result of Prism Excitation. Telecommun. Radio Eng..

[19] Maier S.A. (2007). Plasmonics: Fundamentals and Applications.

[20] Treacy M.M.J. (2002). Dynamical diffraction explanation of the anomalous transmission of light through metallic gratings. Phys. Rev. B.

[21] Wang S.S., Magnusson R. (1993). Theory and applications of guided-mode resonance filters. Appl. Opt..

[22] Dobrowolski J.A. (1995). Optical properties of films and coatings. Handbook of Optics I.

